# Treatment Outcomes of Tuberculosis at Asella Teaching Hospital, Ethiopia: Ten Years’ Retrospective Aggregated Data

**DOI:** 10.3389/fmed.2018.00038

**Published:** 2018-02-21

**Authors:** Ketema Tafess, Teresa Kisi Beyen, Adugna Abera, Geremew Tasew, Shimelis Mekit, Solomon Sisay, Legesse Tadesse, Gilman K. H. Siu

**Affiliations:** ^1^Department of Health Technology and Informatics, The Hong Kong Polytechnic University, Hong Kong, Hong Kong; ^2^Department of Medical Laboratory, College of Health Sciences, Arsi University, Asella, Ethiopia; ^3^Department of Public Health, College of Health Sciences, Arsi University, Asella, Ethiopia; ^4^Leishmaniasis Research Laboratory, Ethiopia Public Health Institute, Addis Ababa, Ethiopia; ^5^Department of Pharmacy, College of Health Sciences, Arsi University, Asella, Ethiopia; ^6^Department of Medical, GLRA-Ethiopia, Addis Ababa, Ethiopia

**Keywords:** tuberculosis, Asella, outcomes, smear-positive pulmonary tuberculosis, smear-negative pulmonary tuberculosis

## Abstract

**Background:**

Directly Observed Treatment Short-course (DOTS) has been one of the major strategies to combat the epidemic of tuberculosis (TB) globally. This study aimed to evaluate TB treatment outcomes between September 2004 and July 2014 under the DOTS program at one of the largest public hospitals in Ethiopia.

**Methods:**

A retrospective data of TB patients registered at Asella Teaching Hospital between September 2004 and July 2014 were obtained from hospital registry. Treatment outcomes and types of TB cases were categorized according to the national TB control program guideline. Binomial and multinomial logistic regression models were used to analyze the association between treatment outcomes and potential predictor variables.

**Results:**

A total of 1,755 TB patients’ records were included in the study. Of these, 945 (53.8%) were male, 480 (27.4%) smear-positive TB, 287 (16.4%) HIV positive, and 1,549 (88.3%) new cases. Among 480 smear-positive pulmonary TB cases, 377 (78.5%) patients were cured, 21 (4.40) completed the treatment, 35 (7.3%) transferred out, 19 (4.0%) died, 24 (5.0%) defaulted, and 4 (0.8%) failure. Overall, 398 (82.9%) smear-positive pulmonary TB patients were successfully treated. For smear-negative TB (*n* = 641) and extrapulmonary TB cases (*n* = 634), 1,036 (81.3%) completed the treatment and demonstrated favorable response. Taking all TB types into account, 1,434 (81.7%) were considered as successfully treated. In the multivariate binary logistic model, patients in older age group (AOR = 0.386, 95% CI: 0.250–0.596) and retreatment cases (AOR = 0.422, 95% CI: 0.226–0.790) were less likely to be successfully treated compared to younger and new cases, respectively. In multinomial logistic regression, age increment by 1 year increased the risk of death and default of TB patients by 0.05 (adjusted β = 0.05; 95% CI: 0.03, 0.06) and 0.02 (adjusted β = 0.02; 95% CI: 0.01, 0.04). The odds of TB patients who died during treatment were higher among HIV-infected TB patients (adjusted β = 2.65; 95% CI: 1.28, 5.50).

**Conclusion:**

The treatment success rate of TB patients was low as compared to the national target. TB control needs to be strengthened for the enhancement of treatment outcome.

## Introduction

Tuberculosis (TB) remains a major global health problem and has replaced HIV as the leading cause of death from a single infectious agent in 2016 (1). In 2015, an estimated of 10.4 million people developed TB and 1.4 million died from the disease, 0.4 million of whom were HIV-infected (2).

In absence of appropriate treatment, TB mortality rate remains high (3, 4). Study on the natural history of TB indicated that up to 70% of smear-positive and 20% of smear-negative cases of pulmonary TB died within 10 years if patients remain untreated (5). Cognizant of this fact, World Health Organization (WHO) introduced Directly Observed Treatment Short-course (DOTS) strategy in the early 1990s, with the target to cure 85% of newly detected cases of sputum smear-positive TB and to detect 70% of the estimated incidence of sputum smear-positive TB (6, 7).

In Ethiopia, DOTS program was implemented in 1992 as a pilot in Arsi and Bale Zones of Oromia Region (8). The program had been subsequently scaled up throughout the country and implemented at national level. According to Federal Ministry of Health assessment in 2013/2014, DOTS program physical coverage reached 98.4% in hospitals and 79% health centers in the country (9). Nevertheless, different reports across the country have indicated the existence of challenges in treatment outcomes of TB patients despite the implementation of DOTS program (10, 11). Poor treatment-seeking behavior, incomplete treatment or poor compliance, treatment interruption, or default, a phenomenon that contributes to prolonged infectiousness and a higher risk of drug resistance, relapsed TB, and death, are the major challenges in DOTS program especially in resource-limited settings (12).

In this study, we aimed to evaluate the treatment outcomes of TB between September 2004 and July 2014 under DOTS program at Asella Teaching Hospital (ATH) in Ethiopia, East African country with TB incidence of 191/100,000 population (2). This hospital was selected because it was one of the first clinical centers that implement DOTS program in Ethiopia, and it has been the only referral hospital in Arsi Zone, serving a large population of approximately 3.5 million, allowing it to best reflect the effectiveness of the DOTS in TB control in the country. The findings also led to the identification of risk factors that are associated with poor treatment outcomes in the current setting. To the best of our knowledge, this is the first and most comprehensive study that specifically addressed the treatment outcome of TB and associated risk factors under DOTS strategy in this public hospital.

## Materials and Methods

### Study Area

Asella Teaching Hospital is University hospital of the newly founded Arsi University, situated in Asella Town, South-East of Addis Ababa. The hospital has 297 beds and acts as a medical referral center for a population of 3.5 million inhabitants in the Arsi Zone and its surroundings. In the hospital, there is one DOTS clinic which has been operating as per National TB and Leprosy Control Program (NTLCP) guideline of Ethiopia. Currently, the clinic provides basic treatment and diagnostic service for all forms of TB through clinical examination, Ziehl–Nielsen staining method for acid-fast bacilli (AFB)/or auramine O-stained sputum smears examination by light-emitting diodes microscopy and chest radiographs. Patients diagnosed with TB were registered and treated according to the national TLCP guideline (8). The DOTS clinic is one of the outpatient clinics of Asella Hospital and the hospital has also isolated rooms for inpatients. Though the hospital provides service for a large number of TB patients per year, the data included in this study, however, is collected from those patients who had complete information registered as per the WHO and national tuberculosis guidelines and comprises the variables included for this study (see next section). All TB patients who had complete information according to the aforementioned guidelines were included in this study.

### Study Design, Period, and Data Collection

Institutional-based retrospective cross-sectional study was conducted. Treatment outcome of all TB patients registered between September 1, 2004, and July 28, 2014, at DOTS clinic of the Asella Hospital were considered as a source of data for investigation. Registered data with complete information were collected retrospectively by trained health professionals using a structured checklist. The registration logbooks were reviewed for basic information such as patient’s age, sex, date of treatment started, date of treatment completed, the address of patients, HIV serostatus, type of TB cases, and treatment outcome.

### Case and Treatment Definitions

Following the standard practices in Asella Hospital, referral or other patients visiting the health facility are preliminary screened at the triage and directed to the physician. After detailed physical examination, patients with presumptive of pulmonary TB are directed to TB diagnostic laboratory. Accordingly, the patients are categorized as a case of smear-positive pulmonary TB if at least one of the two sputum specimens are positive for AFB microscopy, and chest radiographic abnormalities consistent with active pulmonary TB. The patient is categorized as smear-negative TB if at least two sputum smear examinations were negative for AFB on different occasions in whom chest radiographic abnormalities and clinical findings are consistent with active pulmonary TB, or had the previous history of TB treatment. While the case of extrapulmonary TB (EPTB) is TB of organs other than lungs. Treatment of TB patients depends on age and weight of the patients and the chemotherapeutic combination will be decided by the physician. In general, the four drug combination therapy is recommended by the Ethiopian national tuberculosis and leprosy control guideline (8). For the new TB cases, patient is treated with a combination of isoniazid (H), rifampicin (R), pyrazinamide (P), and ethambutol (E) for the first 2 months’ (2RHZE) intensive phase and rifampicin and isoniazid for 4 months of continuation phase (4RH) whereas in retreatment cases streptomycin is also added to the aforementioned combination for the intensive phase, and ethambutol is added to the continuation phase and the continuation period is extended to 5 months.

### Definitions Used in This Study

Treatment outcome definitions were based on the NTLCP (8) and WHO guidelines (6) as indicated in (Table 1).

**Table 1:** Definitions of treatment outcome used in this study.

Indicator	Definition
Cured	– A pulmonary TB patient, who was bacteriologically confirmed by acid-fast bacilli (AFB) microscopy at the beginning of treatment, becomes AFB smear negative or culture-negative in the last month of treatment completion and on at least one previous occasion
Completed treatment	– A TB patient who completed the first line treatment course (2RHZE + 4RH) with a favorable response, based on improved radiographic result and/or relief of TB-related clinical symptoms. It was used as an indicator of successfully treatment outcome in smear-negative pulmonary TB and ETB patients
Died	– A TB patient who died from any cause during treatment
Failed	– A smear-positive TB patient whose sputum smear or culture remains positive, or a smear-negative TB/ETB patient who fails to have favorable clinical response at month 5 or later during treatment
Successfully treated	– A patient who was cured or have favorable clinical response when treatment is completed
Default	– Patient who was on treatment for 1 month and interrupted for at least two consecutive months
Transfer out	– Patient who has been transferred to another clinical for treatment and final treatment outcome is unknown
Treatment success rate	– For AFB smear-positive cases, it referred to the percentage of patients who were cured (bacteriologically confirmed by AFB smear) plus who had developed favorable clinical response when treatment is completed among the cases notified to the national health authorities during a specified period. – For AFB smear-negative and EPTB cases, it referred to the percentage of patients who had developed favorable clinical response when treatment is completed among both forms TB cases notified to the national health authorities during a specified period.

### Data Quality Assurance

Data were collected after training was given to the data collectors and supervisors. The pretest was done for checklists at Adama Hospital, which is located 75 km away from Asella Hospital. Double data entry was done to Epi-info version 7 to check the consistency.

### Ethical Statement

Ethical approval was obtained from Arsi University College of Health Ethical Review Board (Reference number A/U/H/C/87/4016) and support letter was also obtained from medical director office of Asella Hospital.

### Statistical Analysis

Data were cleaned, edited and entered into Epi Info version 7, and then transferred to STATA version 12 for further analysis. Descriptive analysis was done for most of the variables. Binomial and multinomial logistic regression analyses were done to identify factors associated with treatment outcomes. Odds ratio with 95% confidence interval and *P*-values at <0.05 were used to fit the final model. Model fitness for multinomial regression was checked by likelihood ratio test which indicates good model fit.

## Results

### Sociodemographic Characteristics

Complete registration information was obtained from a total of 1,755 TB patients at the TB Clinic of Asella Hospital between September 01, 2004, and July 28, 2014. Out of these, 945 (53.8%) were male and 1,548 (88.2%) rural resident. The median age of the patients was 25 years with interquartile range of 15 and median age of 25 years, and age ranged from 0.59 up to 95 years. HIV co-infection was recorded in 287 (16.4%) of the TB patient while 294 (16.8%) have undetermined HIV serostatus. Majority, 1,549 (88.3%) of the registered TB cases were new patients. The overall classification of TB patients by the type of TB indicated that 480 (27.40%) were smear-positive pulmonary TB, 641 (36.50%) smear-negative pulmonary TB, and 634 (36.1%) EPTB (Table 2).

**Table 2:** Sociodemographic characteristics and status of TB patients registered between September 01, 2004, and July 28, 2014, at Asella Hospital, Arsi Zone, Oromia Region, Ethiopia (*n* = 1,755).

Characteristics	Number	Percent
**Gender**		
Male	945	53.8
Female	810	46.2

**Age (years)**		
<25	924	52.6
25–34	334	19.0
35–44	225	12.8
45–54	137	7.8
≥55	135	7.7

**Residence**		
Rural	1,548	88.2
Urban	207	11.8

**HIV status**		
HIV positive	287	16.4
HIV-negative	1,174	66.9
HIV not tested	294	16.8

**TB type**		
Pulmonary smear positive	480	27.4
Pulmonary smear negative	641	36.5
Extra pulmonary	634	36.1

**Category of TB**		
New	1,549	88.3
Retreatment	53	3.0
Others^a^	87	5.0
Transfer in	66	3.8

*^a^Relapse and treatment after default*.

### Treatment Outcome of Smear-Positive TB

According to the national TB control guideline, acid-fast staining is performed for smear-positive pulmonary TB patients under treatment at three different points: at the end of the 2 months’ intensive treatment phase, at 5th months during treatment, and at the end of 6th or 8th months to declare that the patient is cured or relapsed (8). Of the total 480 smear-positive TB patients who had complete information and registered during the study period, 377 (78.5%) were acid-fast staining negative at the end of the treatment phase and on at least one previous occasion.

The evaluation of treatment outcome of smear-positive pulmonary TB showed that 377 (78.5%) patients were cured, 21(4.4%) completed the treatment, 35 (7.3%) transferred out to another health facility, 19 (4.0%) died, 24 (5.0%) defaulted, and 4 (0.8%) treatment failure. Low cure rate [47 (75.8%)] and high death rate [6 (9.7%)] were observed in TB patients co-infected with HIV. The proportion of defaulter was high [3 (12.5%)] in TB patients older than 55 years (Table 3).

**Table 3:** Treatment outcome of smear-positive TB patients by sociodemographic and clinical factors among patients registered between September 01, 2004, and July 28, 2014, at Asella Hospital, Arsi Zone, Oromia Region, Ethiopia (*n* = 480).

Characteristics	Total	Treatment outcomes					
		Cured	Failure	Died	Completed	Defaulted	Transferred out
**Gender**							
Male	248	183 (73.8)	0	10 (4.0)	15 (6.0)	18 (7.3)	22 (8.9)
Female	232	194 (83.6)	4 (1.7)	9 (3.9)	6 (2.6)	6 (2.6)	13 (5.6)

**Age (years)**							
<25	292	237 (81.2)	0	6 (2.1)	16 (5.5)	13 (4.5)	20 (6.8)
25–34	89	67 (75.3)	2 (2.2)	6 (6.7)	1 (1.1)	2 (2.2)	11 (12.4)
35–44	48	39 (81.3)	0	3 (6.3)	1 (2.1)	4 (8.3)	1 (2.1)
45–54	27	18 (66.7)	0	2 (7.4)	3 (11.1)	2 (7.4)	2 (7.4)
≥55	24	16 (78.5)	2 (8.3)	2 (8.3)	0	3 (12.5)	1 (4.2)

**HIV status**							
Negative	335	265 (79.1)	1 (0.3)	10 (3.0)	17 (5.1)	14 (4.2)	28 (8.4)
Positive	62	47 (75.8)	2 (3.2)	6 (9.7)	1 (1.6)	3 (4.8)	3 (4.8)
Not tested	83	65 (78.3)	4 (0.8)	3 (3.6)	3 (3.6)	7 (8.4)	4 (11.4)

**Category of patients**							
New	398	322 (80.9)	3 (0.8)	9 (2.3)	19 (4.8)	20 (5.0)	25 (6.3)
Retreatment	45	27 (60.0)	0	8 (17.8)	1 (2.2)	2 (4.4)	7 (15.6)
Others^a^	4	2 (50.0)	0	2 (50.0)	0	0	0
Transfer in	33	26 (78.8)	1 (3)	0	1 (3)	2 (6.1)	3 (9.1)
Total	480	377 (78.5)	4 (0.8)	19 (4.0)	21 (4.4)	24 (5.0)	35 (7.3)

*^a^Relapse and treatment after default*.

### Treatment Outcome of Smear Negative and Extrapulmonary (Combined) TB

In the TB clinic of the Asella Hospital, treatment outcomes of any form of TB were registered according to the WHO (13) and national guidelines (8). As stated in the guidelines, the cure rate is calculated for pulmonary TB with bacteriologically confirmed TB at the beginning of treatment who turns negative in the last months of treatment. Consequently, the cure rate was not calculated for EPTB and smear-negative pulmonary TB. Of 1,275 EPTB and smear-negative pulmonary TB patients’ who had complete registration, 1,036 (81.3%) were completed the treatment with favorable responses, 83 (5.4%) transferred out to another health facility, 78 (6.8%) died, 69 (5.0%) defaulted, and 4 (0.8%) treatment failure (Table 4).

**Table 4:** Treatment outcome of combined smear-negative and extrapulmonary TB patients by sociodemographic and clinical factors among patients registered between September 01, 2004, and July 28, 2014, at Asella Hospital, Arsi Zone, Oromia Region, Ethiopia (*n* = 1,275).

Characteristics	Total	Treatment outcomes							
		Completed	Died	Defaulted	Transferred out
**Gender**					
Male	697	561 (80.5)	52 (7.5)	39 (5.6)	45 (6.5)
Female	578	475 (82.2)	35 (6.1)	30 (5.2)	38 (6.6)

**Age (years)**					
<25	632	555 (87.8)	6 (9.0)	27 (4.3)	44 (7.0)
25–34	245	192 (75.3)	22 (6.7)	13(5.3)	18 (7.3)
35–44	177	126 (71.2)	32 (18.1)	10 (5.6)	9 (5.1)
45–54	110	82 (74.5)	14 (12.7)	8 (7.3)	6 (5.5)
≥55	111	81 (78.5)	13 (11.7)	11 (9.9)	6 (5.4)

**HIV status**					
Negative	839	697 (83.1)	39 (4.6)	42 (5.0)	61 (7.5)
Positive	225	181 (80.4)	21 (9.3)	10 (4.4)	13 (5.8)
Not tested	211	158 (74.9)	27 (12.8)	17 (8.1)	9 (4.3)

**Category of patients**					
New	1,151	934 (81.1)	78 (6.8)	64 (5.6)	75 (6.5)
Retreatment	8	7 (87.5)	1 (12.5)	0	0
Others^a^	83	66 (79.5)	7 (8.4)	4 (4.8)	6 (7.2)
Transfer in	33	29 (87.9)	1 (3.0)	1 (3.0)	2 (6.1)
Total	1,275	1,036 (81.3)	87 (6.8)	69 (5.0)	83 (5.4)

*^a^Relapse and treatment after default*.

### Treatment Outcome of All Form of TB

Of 1,755 TB patients who had complete information and registered during the study period, 1,434 were successfully treated, 118 (6.7%) transferred out to another health facility, 106 (6%) died, 95 (5.3%) defaulted, and 4 (0.2%) treatment failure. The highest proportion of successfully treated rate [808 (87.4%)] was observed in patients younger than 25 years and the lowest successfully treated rate [97 (71.9%)] in patients older than 55 years of age. TB patients co-infected with HIV had lower successfully treated rate of 79.8 vs 83.4% and high death rate (9.4 vs 4.2%) when compared with HIV-negative patients. The proportion of defaulter was high [14 (10.4%)] in TB patients older than 55 years (Table 5).

**Table 5:** Treatment outcome of all forms of TB patients by sociodemographic and clinical factors among patients registered between September 01, 2004, and July 28, 2014, at Asella Hospital, Arsi Zone, Oromia Region, Ethiopia (*n* = 1,755).

Characteristics	Total	Treatment outcomes				
		Successfully treated^b^	Failure	Died	Defaulted	Transferred out
**Gender**						
Male	945	759 (80.3)	0	62 (6.6)	57 (6.0)	67 (7.1)
Female	810	675 (83.3)	4 (0.5)	44 (5.4)	36 (4.4)	51 (6.3)

**Residence**						
Rural	1,548	1,265 (81.7)	4 (0.3)	93 (6.0)	74 (4.8)	112 (7.2)
Urban	207	169 (81.6)	0	13 (6.3)	19 (9.2)	6 (2.9)

**Age (years)**						
<25	924	808 (87.4)	0	12 (1.3)	40 (4.3)	64 (6.9)
25–34	334	260 (77.8)	2(0.6)	28 (8.4)	15 (4.5)	29 (8.7)
35–44	225	166 (73.8)	0	35 (15.6)	14 (6.2)	10 (4.4)
44–54	137	103 (75.2)	0	16 (11.7)	10 (7.3)	8 (5.8)
≥55	135	97 (71.9)	2 (1.5)	15 (11.1)	14 (10.4)	7 (5.2)

**HIV status**						
Positive	287	229 (79.8)	2 (0.7)	27 (9.4)	13 (4.5)	16 (5.6)
Negative	1,174	979 (83.4)	1 (0.1)	49 (4.2)	56 (4.8)	89 (7.6)
Not tested	294	226 (76.9)	1 (0.3)	30 (10.8)	24 (8.2)	13 (4.4)

**Category of patients**						
New	1,549	1,275 (82.3)	3 (0.2)	87 (5.6)	84 (5.4)	100 (6.5)
Retreatment	53	35 (66.0)	0	9 (17.0)	2 (3.8)	7 (13.2)
Others^a^	87	68 (78.2)	0	9 (10.3)	4 (4.6)	6 (6.9)
Transfer out	66	56 (84.8)	1 (10.5)	1 (1.5)	3 (4.5)	5 (7.6)

**Type of TB**						
PTB smear positive	480	398 (82.9)	4 (0.8)	19 (4.0)	24 (5.0)	35 (7.3)
PTB smear negative	641	504 (78.6)	0	54 (8.4)	39 (6.1)	44 (6.9)
Extrapulmonary TB	634	532 (83.9)	0	33 (5.2)	30 (4.7)	39 (6.2)
Total	1,755	1,434 (81.7)	4 (0.2)	106 (6.0)	93 (5.3)	118 (6.7)

*^a^Relapse and treatment after default*.

*^b^Involved patients who were cured (confirmed bacteriologically) and who had completed treatment with favorable response*.

### TB Treatment Success and Associated Factors

The treatment success rates were 82.9, 78.6, and 83.9% for smear-positive, smear negative, and EPTB, respectively. Overall, 1,434 (81.7%) were successfully treated for all forms of TB. The proportion of treatment success decline as the age of the TB patients increases (Table 6). Sociodemographic and other clinical related factors in relation to TB treatment outcome were analyzed by bi-variable and multivariable logistic regression model.

**Table 6:** Crude and adjusted odds ratios for various factors that might affect treatment outcome among tuberculosis patients registered between September 1, 2004, and July 28, 2014, at Asella Hospital, Arsi Zone, Oromia Region, Ethiopia (*n* = 1,755).

Characteristics	Treatment success		Crude OR	95% CI	*P*-value	Adjusted OR	95% CI	*P*-value
	Yes	No						
**Sex**								
Female	675 (83.3)	135 (16.7)	1			1		
Male	759 (80.3)	186 (19.7)	0.816	0.639–1.042	0.104	0.834	0.649–1.071	0.154

**Age (years)**								
<25	808 (87.4)	116 (12.6)	1			1		
25–34	260 (77.8)	74 (22.2)	0.504	0.365–0.697		0.521	0.372–0.730	
35–44	166 (73.8)	59 (26.2)	0.404	0.283–0.576		0.422	0.290–0.612	
44–55	103 (75.5)	34 (24.8)	0.435	0.282–0.671		0.457	0.293–0.711	
≥55	97 (71.9)	38 (28.1)	0.366	0.240–0.559	0.000	0.386	0.250–0.596	0.000

**HIV status**								
Negative	979 (83.4)	195 (16.6)	1			1		
Positive	229 (79.8)	58 (20.2)	0.786	0.567–1.090		1.017	0.715–1.446	
Not tested	226 (76.9)	68 (23.1)	0.662	0.485–0.904	0.024	0.754	0.545–1.043	0.199

**Type of TB**								
Extrapulmonary TB	532 (83.9)	102 (16.1)	1			1		
PTB smear positive	398 (82.9)	82 (17.1)	0.705	0.531–0.937		0.782	0.583–1.048	
PTB smear negative	504 (78.6)	137 (21.4)	0.931	0.677–1.279	0.034	0.989	0.704–1.389	0.188

**Category of TB**								
New	1,275 (82.5)	274 (17.7)	1			1		
Retreatment	35 (66.0)	18 (34.0)	0.418	0.233–0.749		0.422	0.226–0.790	
Others^a^	68 (78.2)	19 (21.8)	0.769	0.455–1.300		0.981	0.570–1.688	
Transfer in	56 (84.8)	10 (15.2)	1.203	0.606–2.388	0.021	1.046	0.519–2.107	0.061

*^a^Relapse and treatment after default*.

In the binary logistic regression model, the proportion of TB patients had unsuccessful treatment outcome varied by age group and treatment category. TB patients in old age (AOR = 0.386, 95% CI: 0.250–0.596) and retreatment cases (AOR = 0.422, 95% CI: 0.226–0.790) were less likely to be successfully treated compared to young and new TB cases, respectively (Table 6).

Multinomial logistic regression fitted to the recorded factors revealed that age of the patient, type of TB cases, and HIV infectivity were associated with TB treatment outcome. Accordingly, an increment of age by 1 year increased the risk of death of TB patients by 0.05 (adjusted β = 0.05; 95% CI: 0.03, 0.06) and increased the risk of treatment default of TB patients by 0.02 (adjusted β = 0.02; 95% CI: 0.01, 0.04) at the time of treatment. The odds of TB patients who died during treatment were higher among HIV-infected patients (adjusted β = 2.65; 95% CI: 1.28, 5.50) (Table 7).

**Table 7:** Factors associated with TB treatment outcome among patients registered from between September 1, 2004, and July 28, 2014, at Asella Hospital, Arsi Zone, Oromia Region, Ethiopia (*n* = 1,755).

Covariates	Cured	TB treatment outcomes							
		Completed		Died		Defaulted		Transferred out	
		COR (95%CI)	AOR (95%CI)	COR (95%CI)	AOR (95%CI)	COR (95%CI)	AOR (95%CI)	COR (95%CI)	AOR (95%CI)
**Category of patients**									
New	1								
Retreatment		0.04 (0.02, 0.13)	**	1.09 (0.50, 2.37)	4.62 (1.76, 12.14)	0.25 (0.06, 1.07)	**	0.74 (0.32, 1.72)	**
Others		1.00 (0.66, 1.52)	**	1.17 (0.56, 2.48)	**	0.85 (0.36, 2.0)	**	1.12 (0.55, 2.31)	**
**HIV status**									
Negative	1								
Positive		1.48 (1.04, 2.10)	**	3.15 (1.80, 5.53)	2.65 (1.28, 5.50)	1.33 (0.67, 2.62)	**	1.03 (0.56, 1.90)	**
Not tested		0.78 (0.57, 1.07)	**	2.23 (1.32, 3.75)	**	1.56 (0.91, 2.68)	**	0.53 (0.28, 1.00)	0.31 (0.14, 0.67)
Age of patients	1	1.01 (1.003, 1.02)	**	1.05 (1.04, 1.07)	1.05 (1.03, 1.07)	1.03 (1.02, 1.05)	1.02 (1.01, 1.04)	1.01 (0.99, 1.02)	**

**Not significant at 95% confidence level NB. Since the frequency of failure was low, it was merged to completed category and explained in terms of completed for the outcome variable.

## Discussion

Recommendations on how to evaluate treatment outcomes using standardized categories have been issued by the WHO in conjunction with the European Region of the International Union Against TB and Lung Disease (14). This would make it possible to recognize and amend failures of the system before the incidence and proportion of TB and resistant isolates rise. This study aimed to assess the aggregate 10 years DOTS strategy-based treatment outcomes at Asella Hospital, which is the first hospital in the country selected to pilot the strategy (15).

The relatively higher proportion TB in male (53.8%) in this retrospective data is in agreement with the study conducted elsewhere (10, 16–18), which could be related to the underutilization of health facility by female patients that could intern be due to various socioeconomic and cultural influences as suggested by Mohammed et al. (16) or could also be due to the real biological differences between male and female in susceptibility to development of active disease (19).

The overall 5.5% defaulter rate in this study is close to 5% global target by WHO (13) but lower than the 15 years average default rate of 8.6% of Arsi Zone (15) and 5 years average default rate of 18.3% from Gondar University Teaching Hospital in North West Ethiopia (20) and 9.8% from a study done in South Africa (16). The lower defaulter rate in this study might be due to the deployment of health extension workers (HEWs) in the community which aimed to deliver selected high impact packages of curative and preventive interventions at the community level in all zones in Ethiopia (21). The presence of strict and continuous absentees tracing practice in the community by the HEWs has significantly reduced the number TB defaulters (9).

Treatment failure is the major threat to the control of TB as these patients are at higher risk of developing drug-resistant TB and often associated with poor retreatment outcome (22). The average treatment failure rate of 0.2% in this study is consistent with 0.2% (95% CI: 0.19%, 0.20%) by Mesay et al. (23), 0.4% (95% CI: 0.26%, 0.59%) by Belete et al. (24), 0.5% (95% CI: 046%, 0.53%) by Fantahun et al. (25), and 0.5% (95% CI: 0.49%, 0.51) by Shallo et al. (15). The lower treatment failure in this study could be related to the higher smear conversion rate of smear-positive pulmonary TB at the end of the intensive treatment phase which is the single most significant predictor of the TB treatment failure (26) that could also be potentially linked to the low drug resistance rate in the area (27).

Mortality due to TB is one of the major grave treatment outcomes and that could be associated with different comorbidities (28). In this study, the average 6% (CI: 4.91%, 7.19%) mortality rate is close to 7.4% (CI: 6.97%, 7.83%) reported by Shallo et al. (15). from Arsi Zone and lower than 10% (CI: 9.20%, 11.07%) reported by Belay et al. (20). from Gondar University Teaching Hospital, and 13.6% (CI: 11.69%, 15.65%) recorded in South Africa by Mohammed et al. (16). As in the case reported by Belay et al. (20), Lee et al. (29), and Süreyya et al. (30), the death rate in our study is higher in older and retreatment TB patients.

A study conducted in Arsi Zone (15) reported average treatment success rate of 83.6% which is comparable to our current finding of 81.7%. The current treatment success rate; however, is lower than the national average of 92.1% for the year 2014/15 for new bacteriologically confirmed PTB cases (31) and 85% treatment success target by WHO (32). On the other hand, our result is higher when compared to similar studies done in North Western part of Ethiopia (20, 25). The higher treatment success rate in our finding than studies from North West of Ethiopia might be due to the high proportion of transfer outpatients in those studies where it was not possible to identify the treatment outcomes of transferred outpatients.

The higher treatment success rate in the younger age group is in agreement with the report from elsewhere (15, 33–35). Older individuals often have concomitant diseases, general physiological deterioration with age, less able to reach health facilities and are also poorer than the younger population (36). Retreatment cases (AOR = 0.422, 95% CI: 0.226–0.790) in comparison with new TB cases were less likely to be successfully treated. This is consistent with a study conducted in Arsi zone of Ethiopia (15), Northern part of Ethiopia (35), and South Africa (16). This might be due to the high chance of MDR-TB among previously treated patients than new TB cases (37).

In multinomial logistic regression, an increment of age by 1 year increased the risk of death of TB patients by 0.05. This might be related to the gradual loss of competent immune response (38), which might increase the risk of death if individual caught TB in their late age (39). On the other hand, an increment of age by 1 year increased the risk of default of TB patients by 0.02. The possible explanation might be linked to the fact that in young age, families/or guardian may give due emphasis for their child to adhere to TB treatment; however, as the age increase, they become independent to decide by themselves which might give less attention to treatment adherence. At a late age, the increased risk of defaulter rate might be due to physiological deterioration, inability to reach physically to health facilities and poor socioeconomic status than the younger population.

Our findings revealed that the odds of having TB patients who died during treatment were higher among HIV-infected patients. This is similar to WHO report for African region but lower than global report where the death rate of TB patients among HIV positive was higher than HIV-negative TB patients (11 vs 3.4%) (13). Our finding is also in agreement with the study from South Africa (40) where they indicated a strong association between HIV infection and mortality due to TB. This indicates further efforts are needed to strengthen the recommended WHO TB/HIV collaborative activities at all level of the health care delivery system.

Based on our findings, to enhance the favorable TB treatment outcomes and strengthen the control efforts, we suggest the following points to be considered: following the current WHO End TB strategy direction (1), focusing on addressing the challenges in defaulters and promoting adherence (involving families and communities) should be deliberated. Early diagnosis of TB including universal drug-susceptibility testing and systematic screening of contacts and high-risk groups and collaborative TB/HIV activities, and management of comorbidities are recommended.

Our report has also some limitations. We conducted a retrospective study to assess the treatment outcomes and associated risk factors of TB patients at Asella Hospital. Thus, it could not be possible to establish the causal relationship between the predictors and the outcome variables. Data were also collected from old TB registration books which contained inaccurate and incomplete information about patients. Therefore, we could not claim that this could be free of selection bias. It was also not possible to conduct the drug-susceptibility test on failed cases. Moreover, due to a limitation in obtaining the data of TB patients during the pre-DOTS period, the assessment of the effectiveness of its strategy could not be best achieved by the current research only and the different categories of EPTB was not available from the registration book.

In conclusion, the overall treatment success is lower than the national target and goal set by WHO. Age and comorbidities are the major predictors of unfavorable treatment outcomes. TB control needs to be strengthened for the enhancement of treatment outcome.

## Author Contributions

KT conceived the idea; KT, TB, SM, LT, and GS designed the study; KT, TB, SM, LT, AA, GT, SS, and GS commented on study design; TB, SM, LT, AA, GT, and SS coordinated the data collection; TB and KT analyzed the data; KT drafted manuscript; KT, TB, SM, LT, AA, GT, SS, and GS participated in the write up and critical review of the manuscript. All authors read and approved the final manuscript.

## Conflict of Interest Statement

The authors declare that the research was conducted in the absence of any commercial or financial relationships that could be construed as a potential conflict of interest.
